# Impact of physical activity on children’s cognitive function and its educational applications: a narrative literature review

**DOI:** 10.3389/fpsyg.2025.1720391

**Published:** 2026-01-12

**Authors:** Hongjin Dong, Shen Wang

**Affiliations:** School of Physical Education and Sports Science, Fujian Normal University, Fuzhou, China

**Keywords:** academic achievement, children’s cognitive function, Eduball training, physical activity, physical education curriculum

## Abstract

**Introduction:**

Physical activity (PA) is widely recognized as an important factor in promoting children’s cognitive function (CF), yet existing research lacks systematic integration into physical education (PE) practice.

**Methods:**

A narrative systematic review was conducted following PRISMA guidelines, drawing on peer-reviewed studies from five electronic databases. Due to heterogeneity in interventions and outcomes, a narrative synthesis was applied.

**Results:**

The reviewed evidence indicates that PA positively influences executive functions, attention, memory, and academic performance in children. Cognitively engaging and moderate-to-vigorous activities show the most consistent benefits, although effects vary with exercise design and intensity.

**Discussion:**

Appropriately designed PA interventions in PE can effectively support children’s cognitive development. Integrating cognitively engaging activities into school curricula may enhance educational outcomes, while future research should refine implementation strategies.

## 1 Introduction

In recent years, with emerging global competition in technology and science, research has increasingly focused on the cognitive development of children, especially in developmental psychology. Cognitive ability is a multifaceted and complex construct that is crucial for long-run human capital, technological, and scientific development (Hunter, 1986; Breit et al., 2024). As an integral part of human capital, cognitive function (CF) underpins an individual’s learning processes, daily functioning, and environmental adaptation. Building on a broad consensus, subsequent research has found that the school-age period is critical for cognitive maturation (Jing et al., 2024; Kohlberg, 1968; Panhelova et al., 2023). As one of the key interventions for developing children’s cognitive ability during this period, physical activity (PA) promotes cognitive growth through multiple mechanisms. These mechanisms not only include direct effects on the brain but also involve indirect pathways such as improving sleep quality. The relevance of this sleep-activity-cognition nexus is strongly supported by evidence across different age groups, including university students (Haddad et al., 2024), thereby strengthening the theoretical framework for its application in school-aged children (Illman et al., 2008; Schmidt et al., 2015; Kliziene et al., 2020). On the one hand, PA enhances serotonin levels, which in turn supports neurogenesis and synaptic plasticity, thereby exerting positive effects on the development of memory, learning, and executive function in early childhood (Jing et al., 2024). On the other hand, the cognitive functions developed through PA lead to improvements in various academic domains, such as performance in mathematics, language skills, and general memory and reasoning abilities (Arboix-Alió et al., 2022; Cenizo-Benjumea et al., 2024).

In this paper, we address the gap between research and educational application by pursuing two key objectives: On the one hand, we aim to elucidate the specific effects of Physical Activity (PA) on children’s Cognitive Functions (CFs); on the other hand, we seek to develop practical strategies for educators to leverage these activities to enhance students’ cognitive abilities. To address these objectives, we investigate two main research questions: (1) What are the precise mechanisms through which PA impacts CFs in children? (2) What are the effective strategies that can be implemented to optimize cognitive development through Physical Education (PE)? Drawing on existing literature, we examine the effects of PA on children’s CFs and explore how to integrate PA into formal education frameworks. Consistent with the methodological rigor proposed by Impellizzeri and Bizzini (2012), we designed a search strategy and quality assessment procedures to enhance the validity and comprehensiveness of our review. For educators, our study provides valuable insights for shaping educational goals and outlines strategies for enhancing cognitive development through physical engagement. Finally, we offer an evaluation of children’s cognitive performance, paving the way for future scholarly inquiries. Importantly, it also proposes methods through which children can improve their cognitive abilities via structured PA.

Our study contributes to two strands of literature. Firstly, it explores the common knowledge regarding the determinants of cognitive ability. Previous studies have discussed the relationship between PA and CFs (Haddad et al., 2024); however, they often fail to focus on integrating these findings into practical educational settings. This paper not only synthesizes mechanistic evidence regarding how PA influences cognitive functions such as executive control, memory, and academic performance but also proposes a series of actionable strategies for curriculum design and teaching practice.

Secondly, compared to previous research that often examined physical activity within a single domain, we synthesize empirical evidence from diverse fields. We make theoretical advancements across multiple dimensions and integrate these previously disparate findings into a cohesive framework, as demonstrated by: First, we incorporate evidence from Contreras-Osorio et al. (2022) on the relationship between physical fitness and executive functions, thereby enriching the theoretical explanation linking physical fitness to CFs. Second, using the sophisticated movement analysis methodology of Kulis et al. (2023), we provide a methodological foundation for investigating the efficacy of Eduball and other tools in enhancing motor development. Third, by integrating D'Isanto’s (2023) research on curriculum barriers with Via et al.'s (2023) theory on exercise intensity, we establish a theoretical foundation spanning both physiological and policy dimensions. Finally, building on Roca et al. (2023), we explore how short-run effects contribute to long-run development.

## 2 Methodology

### 2.1 Search strategy

In this paper, the selection process was conducted in accordance with the Preferred Reporting Items for Systematic Reviews and Meta-Analyses (PRISMA) Statement (Page et al., 2021), incorporating the methodological guidance for systematic reviews proposed by Impellizzeri and Bizzini (2012). We conducted searches across multiple electronic databases on September 8th, 2023, including PubMed/Medline, Scopus, Web of Science, and ERIC (Education Resources Information Center). These databases were selected for their comprehensive coverage of interdisciplinary literature spanning biomedical, psychological, educational, and sports science domains. This approach aims to minimize database-specific bias and to target peer-reviewed articles concerning the effects of PA on CF in children: PubMed/Medline was included to capture clinically oriented studies; ERIC was searched to identify educational interventions and school-based programs; while Scopus and Web of Science provided broad coverage of multidisciplinary research. The search strategy was informed by the retrieval keywords from Antczak et al. (2020), Bakala et al. (2022), Sanders et al. (2021), Impellizzeri and Bizzini (2012), and Sun et al. (2013). The main keywords for this study were translated into English, and the following search string was applied to titles and abstracts.^1^

This systematic review was designed and reported in accordance with the PRISMA (Preferred Reporting Items for Systematic Reviews and Meta-Analyses) guidelines. Literature searches were conducted on September 8, 2023, across five electronic databases: PubMed/MEDLINE, Scopus, Web of Science, ERIC, and PsycINFO. These databases were selected to achieve comprehensive coverage of the multidisciplinary literature relevant to the topic, spanning biomedical, psychological, and educational sciences. The search strategy utilized keywords derived from previous relevant reviews, and the constructed Boolean logic search string was applied to the title and abstract fields. The initial search yielded 1,253 records. After removing duplicates, 1,052 articles were screened based on titles and abstracts, resulting in 32 articles undergoing full-text review.

To assess the methodological quality of the included studies, the Cochrane Risk of Bias tool (RoB 2) was employed. The assessment was conducted independently by two reviewers. The results indicated that, among the 18 ultimately included studies, the risk of bias arising from the randomization process was generally low (11 studies), although 7 studies were judged as having ‘some concerns’ due to insufficient description of the randomization method. Given the nature of physical activity interventions, most studies (14 studies) raised ‘some concerns’ regarding bias due to deviations from intended interventions (blinding of participants and personnel). However, risks associated with missing outcome data and outcome measurement were low in 15 and 16 studies, respectively. No study showed evidence of selective reporting.

Following data extraction, we assessed the heterogeneity among all included studies concerning interventions, comparator conditions, and outcome measurement tools to determine the feasibility of data synthesis. The assessment revealed substantial heterogeneity in intervention types (e.g., aerobic exercise vs. exergames), intensity, duration, and the specific tools used to measure cognitive function, precluding a meaningful quantitative pooling of results. Consequently, the plan to conduct a meta-analysis was abandoned in favor of a structured descriptive synthesis. Specifically, studies will be categorized by the type of physical activity (e.g., acute exercise, chronic exercise) and the primary cognitive domain assessed (e.g., executive function, attention, memory) for a systematic narrative summary.

### 2.2 Inclusion criteria

**Figure 1:**
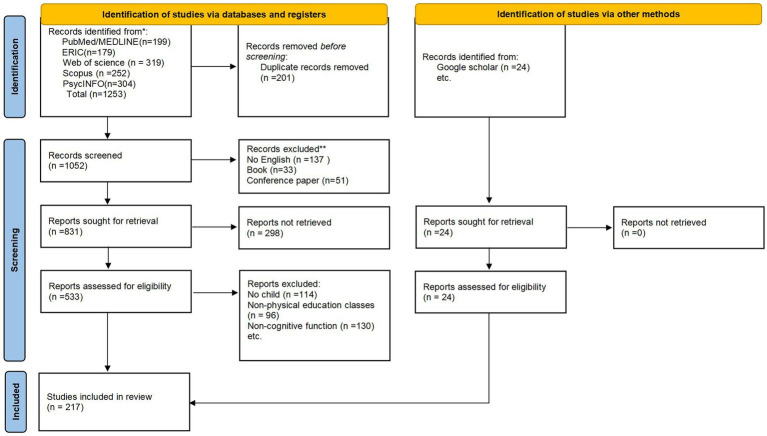
Illustrates the inclusion and exclusion process.

This study implemented a series of inclusion and exclusion criteria. Articles were included if they met the following conditions: (i) crafted in English; (ii) disseminated in a journal subject to peer evaluation.; (iii) focused on children; (iv) described various types of sports activities; and (v) the research question did pertain to CF. After removing duplicates, 1,052 records were screened based on titles and abstracts. A total of 533 articles were selected for full-text review. Following a comprehensive assessment, 114 articles were excluded for not focusing on children, and 226 articles were excluded because their research questions did not pertain to the relationship between physical activity (PA) and cognitive function (CF). Ultimately, 214 articles were included in the final review for data extraction and quality assessment (see Figure 1).

### 2.3 Extraction and analysis

Data were extracted and organized into a Microsoft Excel literature grid with several tabs. The extracted data included author(s), year of publication, country of study, study design, study participants, data collection methods, analysis techniques, and findings.

## 3 Results

This section presents the findings related to two research questions. The first research question investigates the influence of Social–Emotional Learning (SEL) on students’ reading achievement. The second research question examines how teachers incorporate SEL into their teaching to boost students’ reading efficiency.

*Q1:* What is the effect of PA on children’s CF?

CFs consist of a wide range of mental abilities, including memory, attention, executive functions, and learning capacity. Among the many factors that influence CFs, physical activity (PA) is recognized as an important moderator of cognitive development, especially in school-aged children. In this section, we synthesize findings on the effects of PA on CFs. Additionally, based on the literature, we attempt to elucidate the potential mechanisms behind this relationship.

As the core components of cognitive function, Executive Functions (EFs) include inhibitory control, working memory, and cognitive flexibility (Diamond, 2013), which are particularly susceptible to modulation through physical activity (PA). On the one hand, PA involving complex motor tasks, such as those exemplified in elite dance (Kulis et al., 2023), induces functional adaptations within neural networks that support cognitive control, memory, and motor coordination (Ryu et al., 2021). On the other hand, assessing the relationship between physical performance parameters and EFs can provide further insight; for instance, Contreras-Osorio et al.'s (2022) systematic review highlights how specific physical fitness measures correlate with executive function outcomes, reinforcing the interconnectedness of motor and cognitive processes.

PA also benefits other domains, such as mathematical reasoning, language acquisition, and general academic achievement, which extend beyond core EFs. For instance, structured PA interventions have been shown to improve attentional control and memory consolidation, potentially mediated by serotonin-driven neurogenesis and synaptic plasticity (Jing et al., 2024). Moreover, activities requiring fine motor coordination, such as those involving Eduball or tactical game-based tasks, have been linked to gains in visual–spatial processing and task-switching capabilities (Cichy et al., 2022a; Rodríguez-Negro and Yanci, 2022).

However, the literature reveals notable contradictions. While studies report both short- and long-term effects of acute and chronic PA, some findings indicate that high-intensity exercise may transiently impair attention and cognitive performance in children (Mavilidi et al., 2020). These discrepancies may be attributed to contextual factors such as exercise intensity, duration, timing of cognitive assessment, individual differences in baseline fitness, and methodological variations across studies. Furthermore, metabolic responses induced by exercise intensity may represent an important factor influencing cognitive outcomes. For instance, the review by Via et al. (2023) on metabolic factors in tendinopathy (Acta Kinesiologica, 17(2), 62–67), although focused on a different population, provides in-depth insights into the relationship between exercise intensity and metabolic responses.

This study offers a physiological mechanism-based perspective for designing moderate-to-vigorous physical activity (MVPA) programs and helps explain the heterogeneous effects of different exercise intensities on cognitive function. However, the current review provides limited critical evaluation of conflicting findings in the literature. For instance, while it is noted that some studies report potential detrimental effects of acute exercise on attention, this contradiction is not sufficiently explored. Moreover, although the educational applications section offers practical insights, it lacks depth in addressing implementation barriers and contextual factors that may affect the success of interventions. Therefore, it is essential for future research to not only clarify these inconsistencies but also to establish evidence-based parameters for implementing physical activity in educational settings, taking into account such practical and contextual challenges.

*Q2:* How can teachers improve students’ CF in the context of school PE?

### 3.1 Increasing cognitive and physical challenges in PE

Introducing moderate-to-vigorous physical activity (MVPA) and tasks requiring fine motor control can effectively address the cognitive demands of physical education. An 8-week MVPA program incorporating outdoor games has been shown to positively affect children’s cognitive function (Bai et al., 2022). Motor activities involving coordinated movements and hand-eye coordination (Cichy et al., 2022a; Escolano-Pérez et al., 2020) play a critical role in cognitive development. To analyze complex movements, the kinematic analysis framework for elite dance sport competitors proposed by Kulis et al. (2023) (Acta Kinesiologica, 17(1), 60–65) offers a sophisticated methodology that can also be applied to assess and refine coordinated movement and fine motor control tasks, including the use of tools such as Eduball.

To adapt to increasing cognitive and physical challenges in PE, teachers are also encouraged to employ models such as the Tactical Game Model (TGM). Within the TGM framework, students need to execute complex motor skills under dynamic conditions, thereby fostering cognitive flexibility, attention, and creativity (Rodríguez-Negro and Yanci, 2022). Nevertheless, the implementation of such approaches faces practical challenges, including the need for specialized teacher training, adequate equipment, and curricular flexibility. D'Isanto (2023) emphasizes that without systemic support and professional development, even well-designed interventions may encounter implementation barriers.

### 3.2 Reasonable design and implementation of PAs

While PA is beneficial for cognitive development, improper design can lead to diminished returns or even adverse outcomes. The dual-task cost theory highlights that simultaneous cognitive and motor demands may result in performance degradation in one or both domains (Cichy et al., 2022b). Therefore, integrating cognitive tasks with PA must be carefully balanced to avoid overwhelming students. Via et al. (2023) further stress the importance of aligning exercise intensity with metabolic responses to maximize benefits and minimize risks, particularly in MVPA settings. An effective PE curriculum should combine health-enhancing PA, skill-based activities, and periods of reflection, incorporating a variety of sports to maintain engagement and holistic development (Kliziene et al., 2023). Moreover, integrating both linear and nonlinear pedagogical approaches allows for structured skill progression while adapting to individual learning needs (Rudd et al., 2019). However, innovators must avoid overly complex designs that could lead to student fatigue or injury (Kliziene et al., 2020).

### 3.3 Add Eduball training to PE class

Eduball exemplifies an integrated approach that combines motor activities with academic content using printed balls featuring letters, numbers, and symbols (Cichy et al., 2020; Wawrzyniak et al., 2022). This tool promotes collaborative functioning across brain regions, supporting gains in attention, memory, and overall cognitive function. It has also shown promise for children with dyslexia, improving reading and writing skills significantly (Cichy et al., 2022b). The application of fine motor analysis frameworks, such as that proposed by Kulis et al. (2023), can further enhance the implementation and evaluation of Eduball-based interventions. For successful educational integration, teachers are encouraged to tailor Eduball activities to students’ age and curricular needs. However, as highlighted in studies such as D'Isanto (2023) on physical education and educational leadership (Acta Kinesiologica, 17(1), 26–32), such implementation requires not only adequate teacher training and resource allocation but also strategic curriculum alignment and institutional support to overcome common practical challenges.

### 3.4 Attention of schools and family to the development of sports activities

Proper engagement in PA not only augments students’ muscular strength, endurance, flexibility, and coordination but also significantly enhances their CFs (Arboix-Alió et al., 2022). Furthermore, regular participation aids in the management of students’ weight. Contreras-Osorio et al. (2022) suggest that higher adipose-related anthropometric indicators, such as Body Mass Index (BMI) and Waist-to-Hip Ratio (WtHR), are associated with poorer EFs, including working memory and cognitive flexibility. Therefore, it is of utmost importance to sustain regular engagement in PAs.

In addition, Ryu et al. (2021) propose that attention should be paid to the development of PE classes to provide children with high-quality PE resources. Children’s sedentary behavior in the classroom negatively impacts their physical health and CF. In their study, Wick et al. (2018) concluded that reducing sedentary behavior may enhance children’s CF. Similarly, Reisberg et al. (2021) found that children who had higher levels of PA but more sedentary time in kindergarten performed relatively poorly in speech skills as they entered first grade, a finding that underscores the importance of reducing sedentary time to boost children’s cognitive development, particularly in speech skills. Therefore, to increase children’s active time and decrease sedentary time, teachers should organize as many physical recess activities as possible within schools.

Additionally, families play a notable role in shaping the development of children’s PA. Reigal et al. (2019) suggest that families should encourage children to participate in PA to enhance their PF and cognitive abilities. Supporting this, van den Berg et al. (2017) emphasize the importance of collaboration between families, schools, and communities to create more PA opportunities, thereby promoting children’s academic performance and physical health. When considering long-term engagement, factors beyond the immediate social environment also come into play. For instance, Roca et al.'s (2023) study on macroregional differences in cardiorespiratory fitness highlights that broader geographical and socio-cultural contexts can influence sustained participation in PA and its associated cognitive benefits. This underscores the need for a multi-level approach, combining family support with an understanding of larger contextual factors, to foster lasting PA habits and cognitive outcomes in children.

## 4 Conclusion

The primary objective of this study is to explore the effects of PA on children’s CF through a comprehensive literature review and to outline practical strategies for enhancing cognitive development in this demographic. The impetus for this research arises from the apparent absence of an exhaustive review elucidating the benefits of PAs for cognitive development and the methods to optimally foster these functions through sports. Consequently, this article synthesizes activities and curriculum standards that support cognitive development, drawing from pivotal insights and findings within the reviewed literature. It is crucial to recognize that achieving a broad consensus within the academic community may be challenging due to varied conceptual definitions and the ongoing debate over the best strategies to enhance children’s CF. This study advocates for effective collaboration among future researchers, PE teachers, and child guardians, as the development of CFs in children necessitates a scientifically backed approach with consistent implementation. Additionally, it is recommended that schools, families, and communities give due consideration to promoting PA, increasing children’s active time, and collectively fostering cognitive development. This study is limited in its focus primarily on conceptual definitions and, owing to spatial constraints, does not extend the database search. Despite these limitations, this study systematically organizes relevant discussions on the development of children’s CF through PA and may serve as a valuable reference for educators.

## Data Availability

The original contributions presented in the study are included in the article/supplementary material, further inquiries can be directed to the corresponding author.

## Footnotes

1 (TITLE-ABS-KEY (“Primary education” OR “preschool education”)) AND (TITLE-ABS-KEY (“executive functions” OR “memory” OR “attention” OR “cognitive”)) AND (TITLE-ABS-KEY (“Child*” OR “Pupil*” OR “Boy*” OR “Girl*” OR “Preschool*” OR “Toddler*”)) AND (TITLE-ABS-KEY (“Physical activity” OR “physical exercise” OR “exercise” OR “fitness” OR “mind body” OR “Motor (in)activity” OR “Physical (in)activity” OR “Exercis*” OR “Sport*” OR “Acceleromet*” OR “Actigraph*” OR “Physically (in) active” OR “Anaerobic exercise” OR “aerobics” OR “Active transport” OR “active transportation” OR “physical education” OR “lesson” OR “Physical education courses”)).
